# Supplementary material for: Artificial colloidal liquid metacrystals by shearing microlithography
Source: Nat Commun. 2019 Sep 11;10:4111. doi: 10.1038/s41467-019-11941-z (PMC6739410; doi:10.1038/s41467-019-11941-z)
Supplement: Supplementary file 1 — Supplementary Information [file 41467_2019_11941_MOESM1_ESM.pdf]

Supplementary Information

Artificial Colloidal Liquid Metacrystals by Shearing Microlithography

By Jiang *et al.*

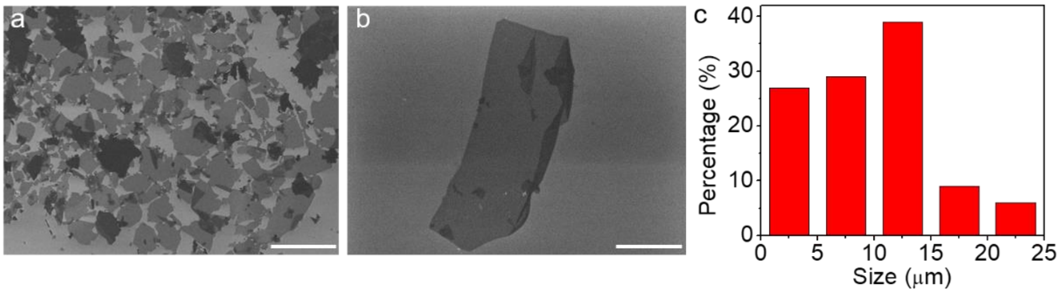

Supplementary Figure 1. Size distribution of GO sheets. a and **b,** Typical SEM image of GO sheets, scale bars, **a**, 20 μm, **b**, 5 μm. **c,** The size distribution of GO sheets.

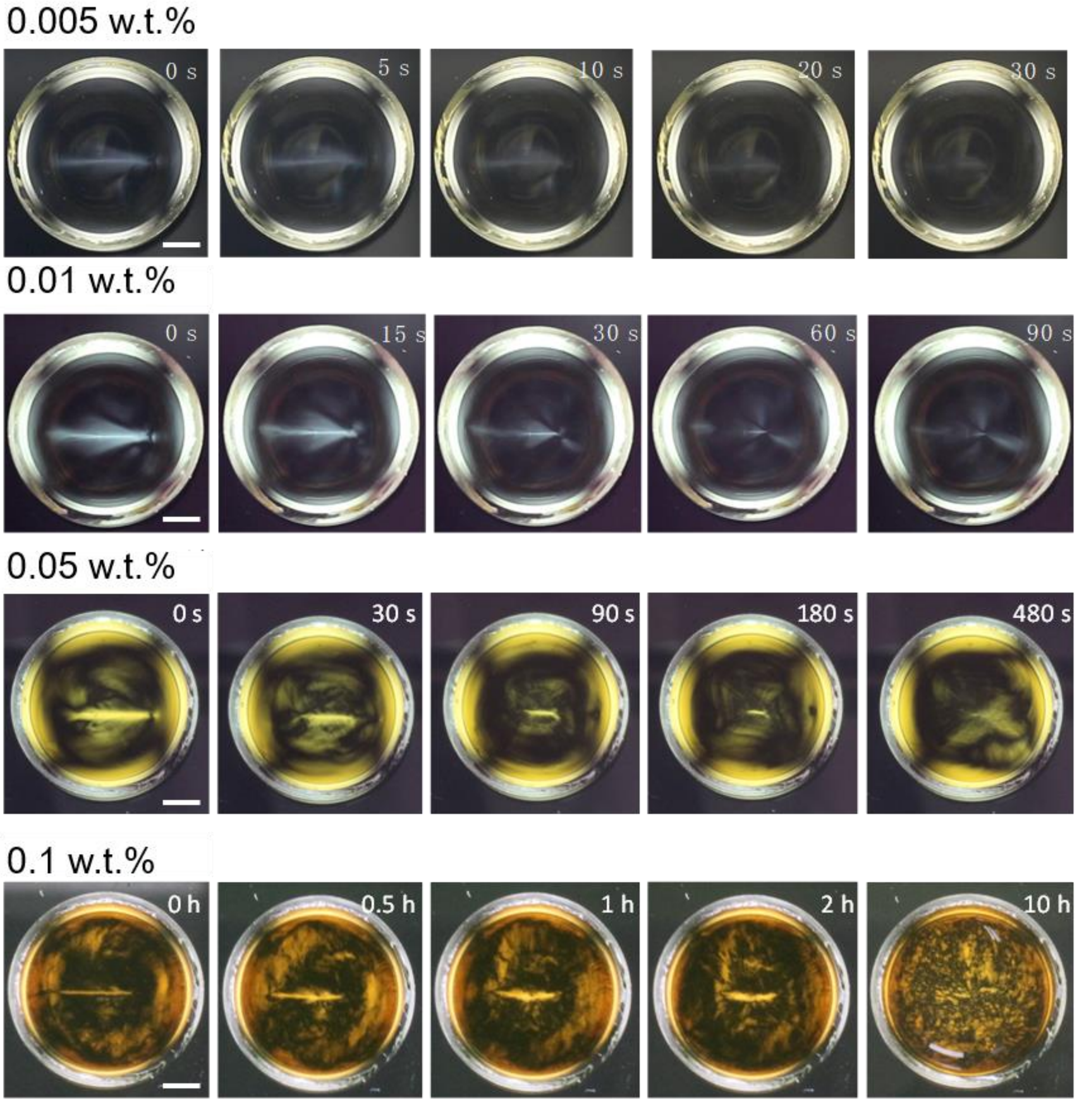

Supplementary Figure 2. The relaxation tracking of GO liquid crystalline grains at different concentrations. To measure the relaxation time of GO LCs, we tracked the wrote birefringent line π-walls under POM and then measured the time ($\boldsymbol{\tau}$) till the birefringence faded away, Scale bars, 2 mm.

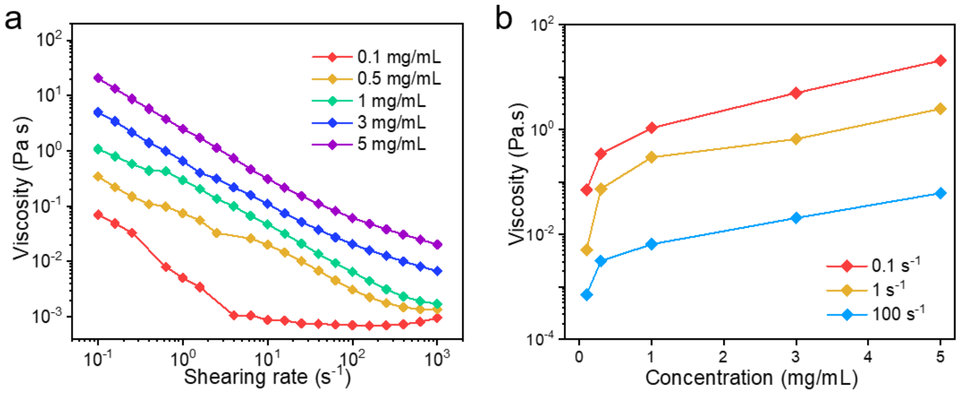

Supplementary Figure 3. The viscosity of GO solutions with different concentrations. a, The steady viscosities of GO solutions with different concentrations at different shear rate. Due to the low mass fraction of GO solutions (< 0.5 wt.%) and the high anisotropy of GO sheets, the GO solutions exhibit a typical shear thinning behavior and relatively low viscosity compared with polymer solutions. In a typical SML process, the moving speed of probe is around 1 – 100 mm s^{-1}, and the diameter of the probe is around 5 – 500 μm. Therefore, the corresponding shear rate of the probe is around 10^0 – 10^4 s^{-1}, in which range, the viscosity is very low. **b,** the steady viscosities as function of GO concentration at shear rate of 0.1, 1 and 100 s^{-1}.

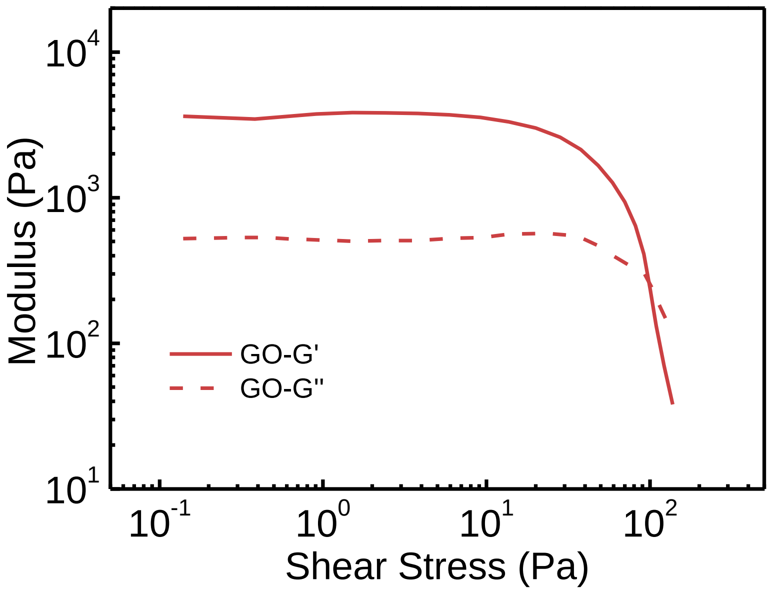

Supplementary Figure 4. The rheological behaviors of GO solution (15 mg mL^{-1}). GO solution displays typical shear-thinning non-Newtonian fluid behavior. Under high shear stress, both G' and G'' decrease drastically, which is favorable for GO sheets to orientate along the direction of shear stress. On the other hand, as the shear stress evacuates, the recovery of the high G' is helpful to retain the orientation.

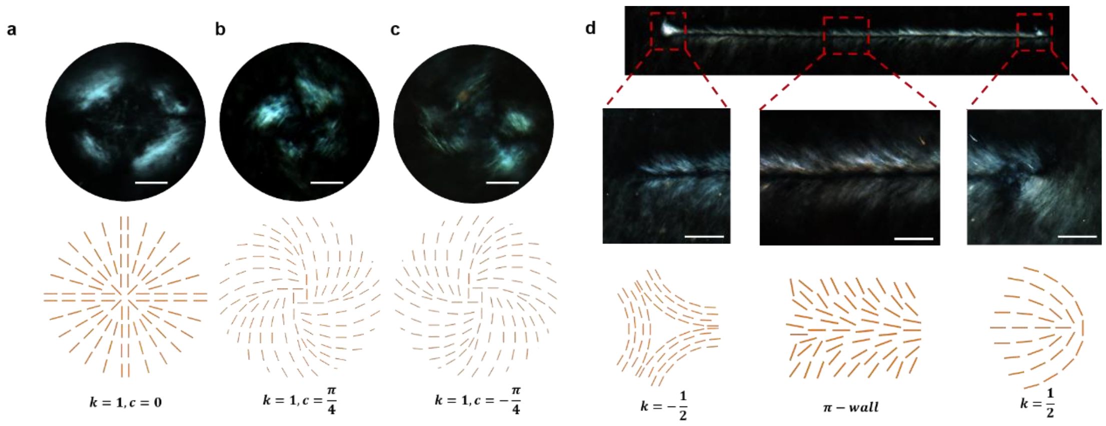

Supplementary Figure 5. Topological structures of defects and basic π-wall of LMCs. a to **c,** Defects with typical integral topological intensity (+1), **b,** the clockwise and **c,** counter-clockwise structures were achieved by constantly rotating the probe. **d,** The drawn π- wall has two topological defects with strength of +1/2 and -1/2 in head and tail, respectively, Scale bars, 20 μm.

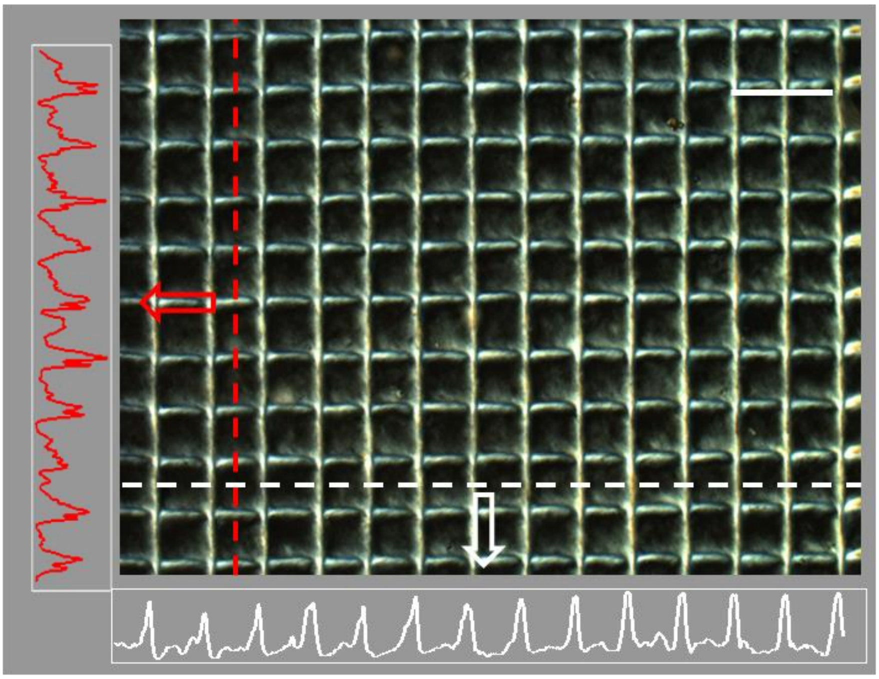

Supplementary Figure 6. Transmitted intensity analyses for LMCs under POM. The transmitted intensity statistics for POM image of cross planes pattern achieved in GO dispersion. The well-matched distribution of peaks implies the well maintained orientation of GO sheets, scale bar, 400 μm.

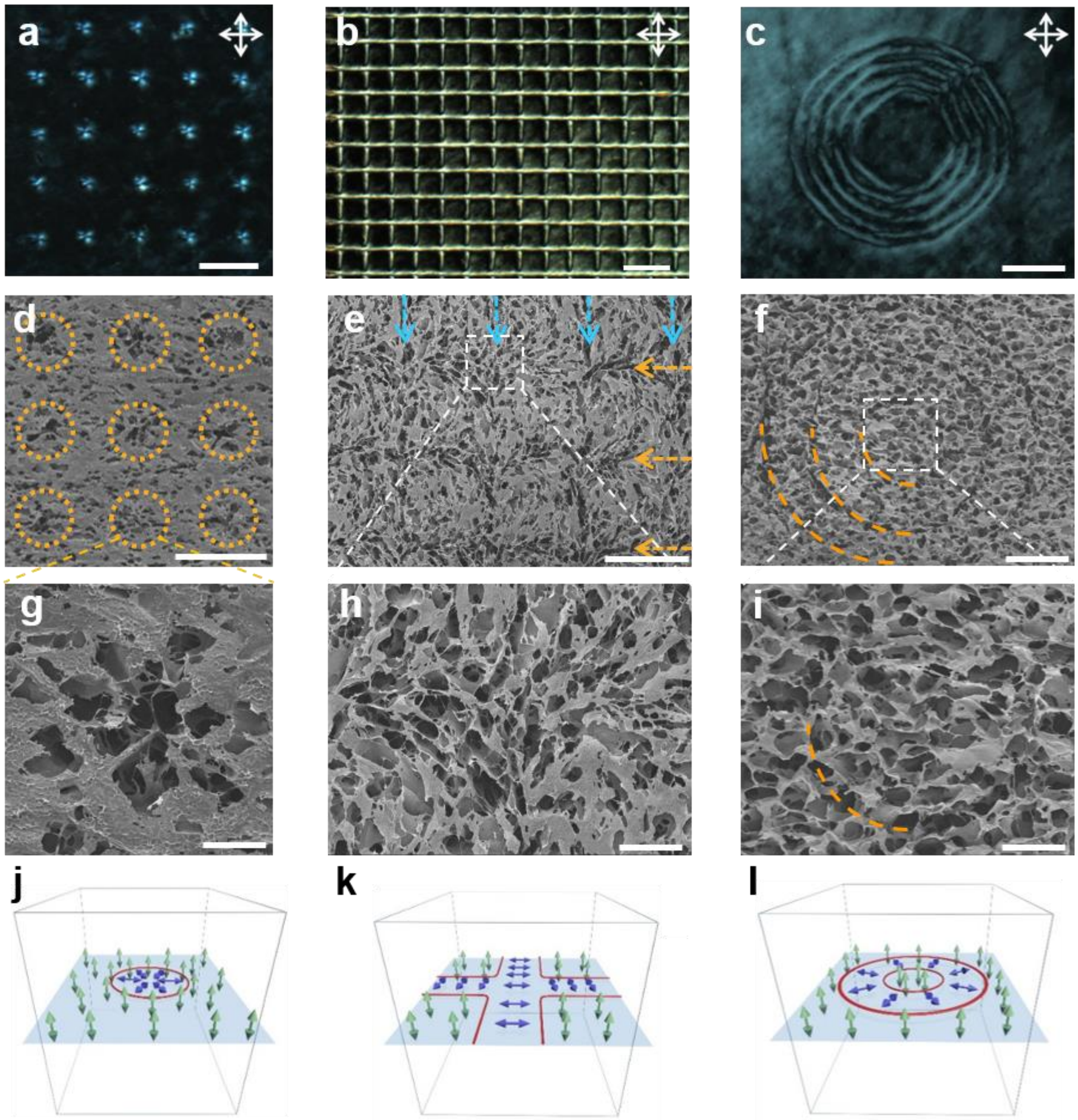

Supplementary Figure 7. Structures of typical LMC by SML. a to **c,** POM images of **a,** typical columns, **b,** cross planes and **c,** concentric surfaces. **d** to **i,** SEM images of **d, g,** typical columns, **e, h,** cross planes and **f, i,** concentric surfaces after quick quenching in liquid nitrogen and freeze drying. **j** to **l,** Top-view of director maps for **j,** typical columns, **k,** cross planes and **l,** concentric surfaces, scale bars, **a, d, e,** 200 μm; **b, c, f,** 400 μm; **g, h,** 50 μm; **i,** 100 μm.

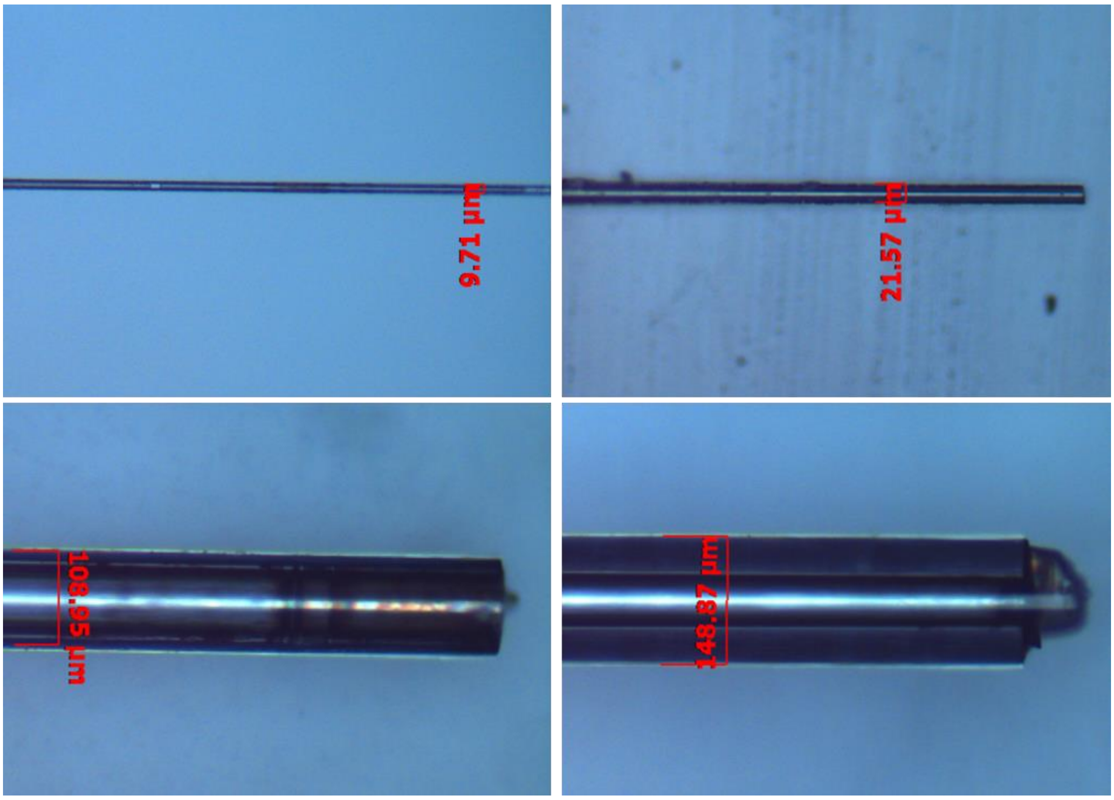

Supplementary Figure 8. Optical microscopy images of micro-probes with diameter ranging from ten to hundreds of micrometers.

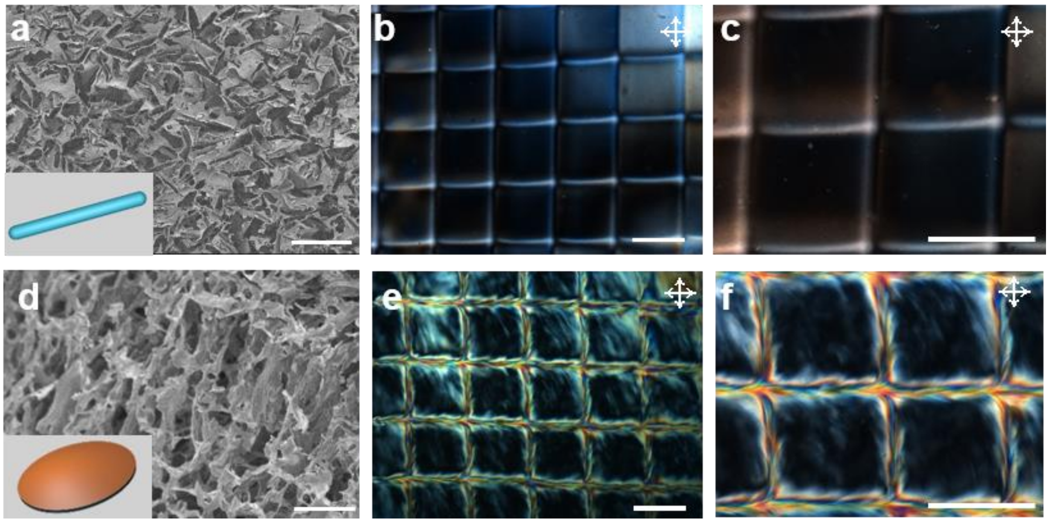

Supplementary Figure 9. LMCs of 1D cellulose nanocrystals and 2D zirconium phosphate. a, SEM image of cellulose nanocrystals (CNCs), a commercial 1D materials. Inset shows the schematic structure of CNCs. **b** and **c,** POM images of crossing patterns in CNC LCs by SML. **d,** SEM image of zirconium phosphate (ZrP), a commercial 2D materials. Inset shows the schematic structure of ZrP. **e** and **f,** POM image of crossing patterns in NP LCs by SML, scale bars, **a, d,** 50 μm; **b, c, e, f,** 200 μm.

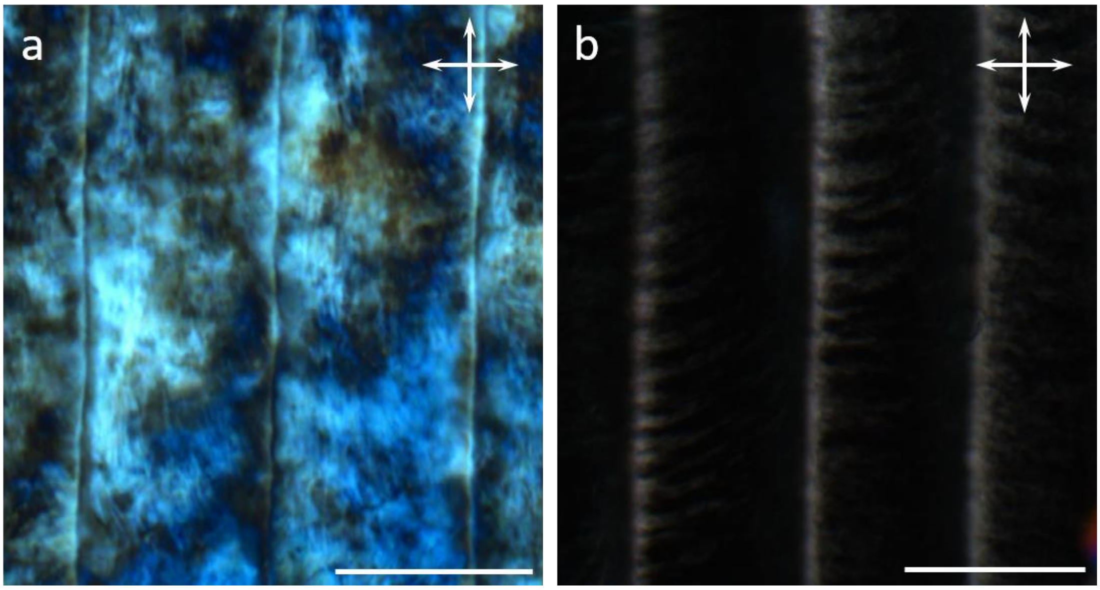

Supplementary Figure 10. LMCs in amphiphilic system. The SML strategy is also available for polymerized lyotropic liquid crystals like nonaethylene glycol monododecyl ether ($C_{12}EO_9$) and cetyltrimethyl ammonium bromide (CTAB), demonstrating its versatility. POM images line patterns in **a,** $C_{12}EO_9$ and **b,** CTAB liquid crystals, scale bar, 200 μm.

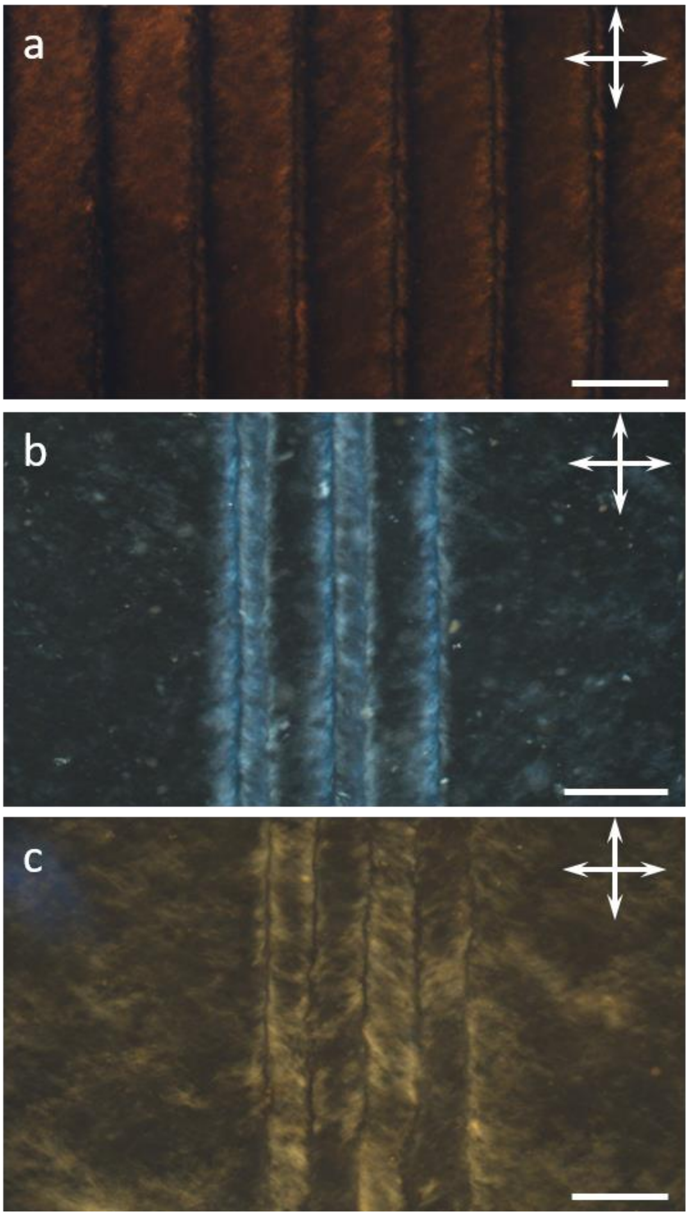

Supplementary Figure 11. LMCs in composite system. We utilize the SML method in GO/polymer composite systems. The POM images display line patterns obtained in **a,** GO/PVA (5 wt.%), **b,** GO/PEG (20 wt.%) and **c,** GO/Glucose (5 wt.%), scale bars, **a,** 200 μm, **b, c,** 400 μm.

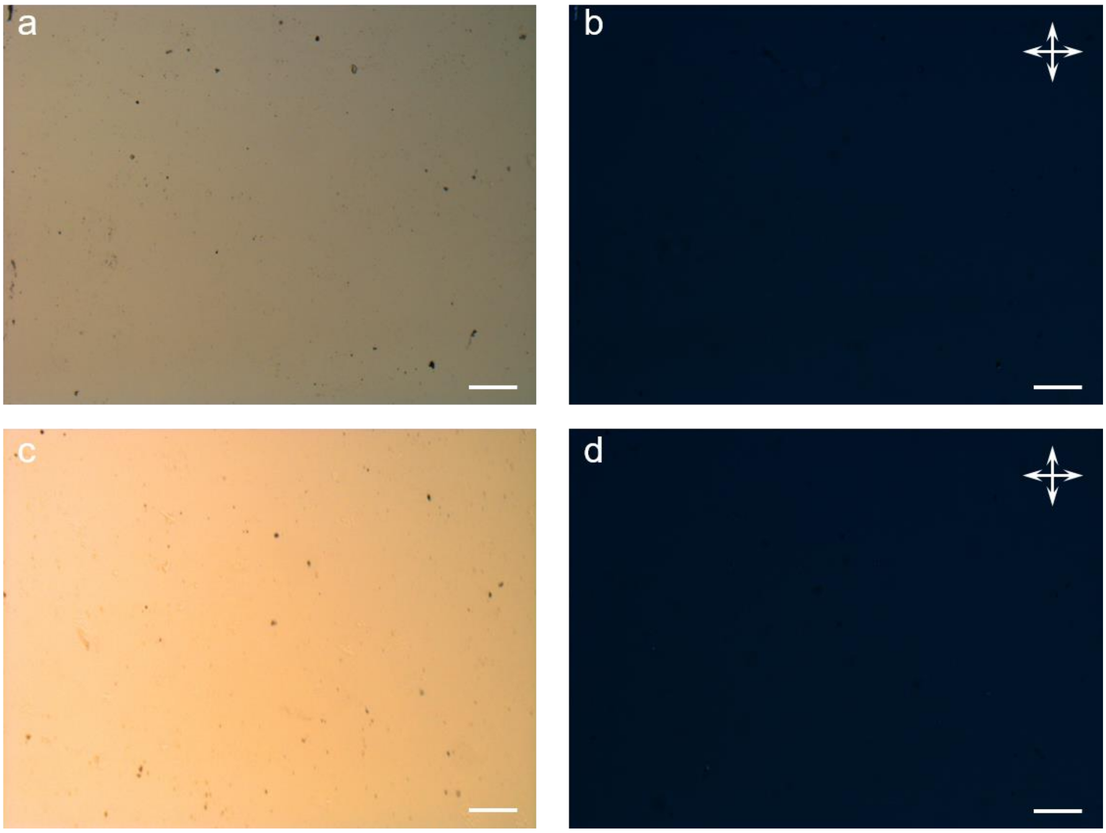

Supplementary Figure 12. SML in polymeric solutions. SML was used to construct laminar structures in (**a**, **b**) 5 wt.% PVA and (**c, d**) 20 w.t.% PEG aqueous solutions. After the SML process, no visible laminar structures were observed under neither (**a, c**) optical microscope nor (**b, d**) polarized optical microscope. Therefore, the SML is only valid in LC systems, Scale bar, 200 μm.

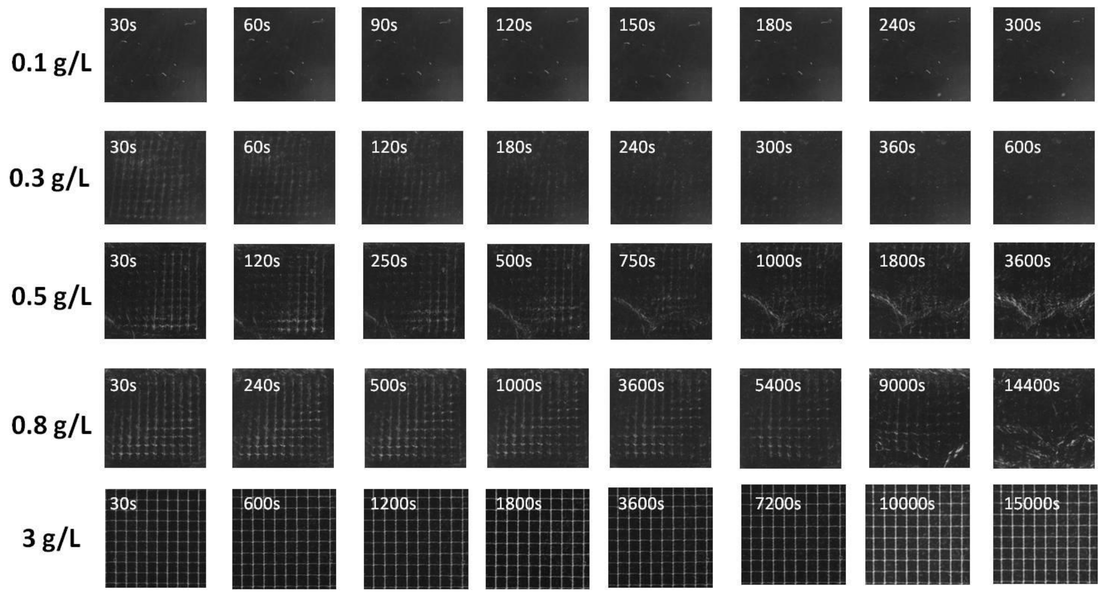

Supplementary Figure 13. Relaxation behavior of LMCs. The POM images for LMCs with different concentrations in the relaxation process.

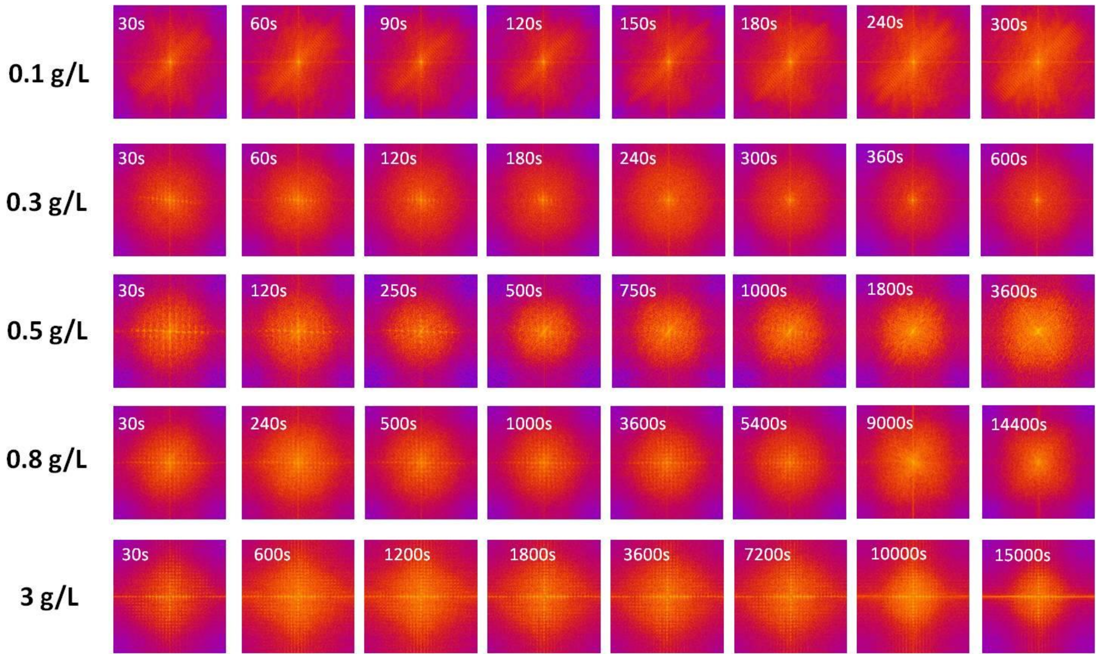

Supplementary Figure 14. Change of *S* in the relaxation of LMCs. The corresponding FFT intensity images for POM images of LMCs with different concentrations in the relaxation process.

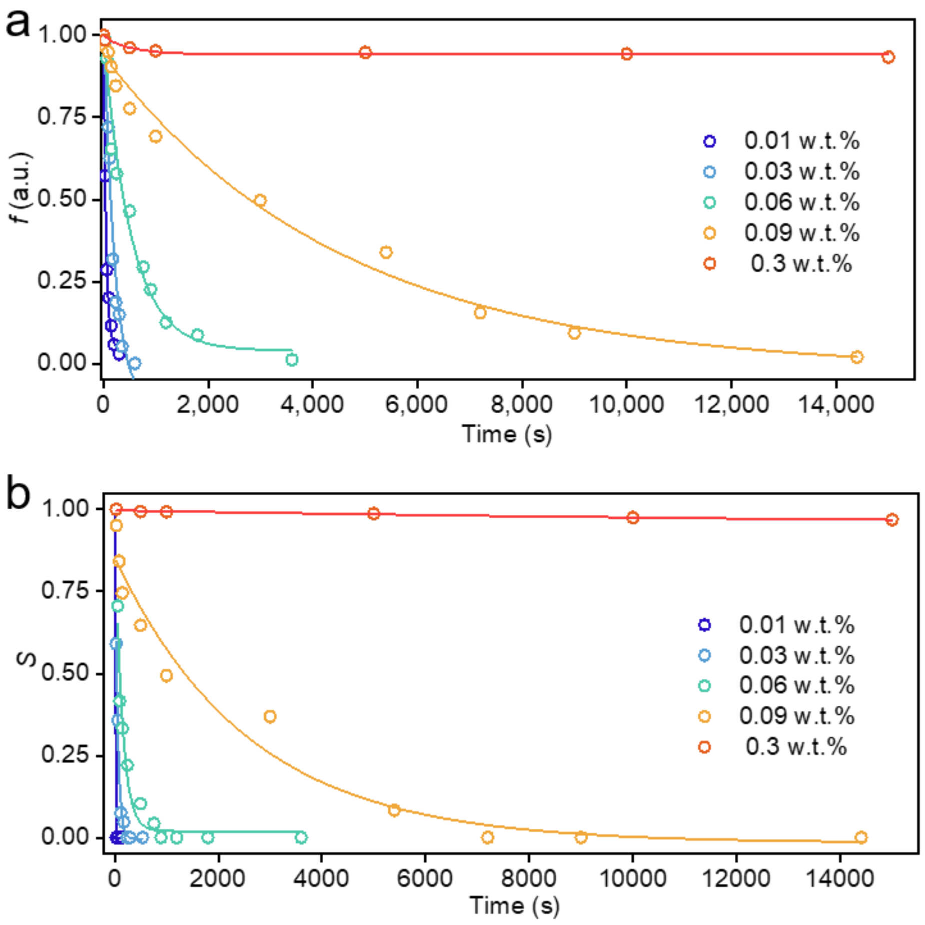

Supplementary Figure 15. Original data of the relaxation kinetics of LMCs. a, The fraction of reoriented domains (*f*) with time (*t*). **b,** *S* as a function of *t* for GO LCs with different concentrations.

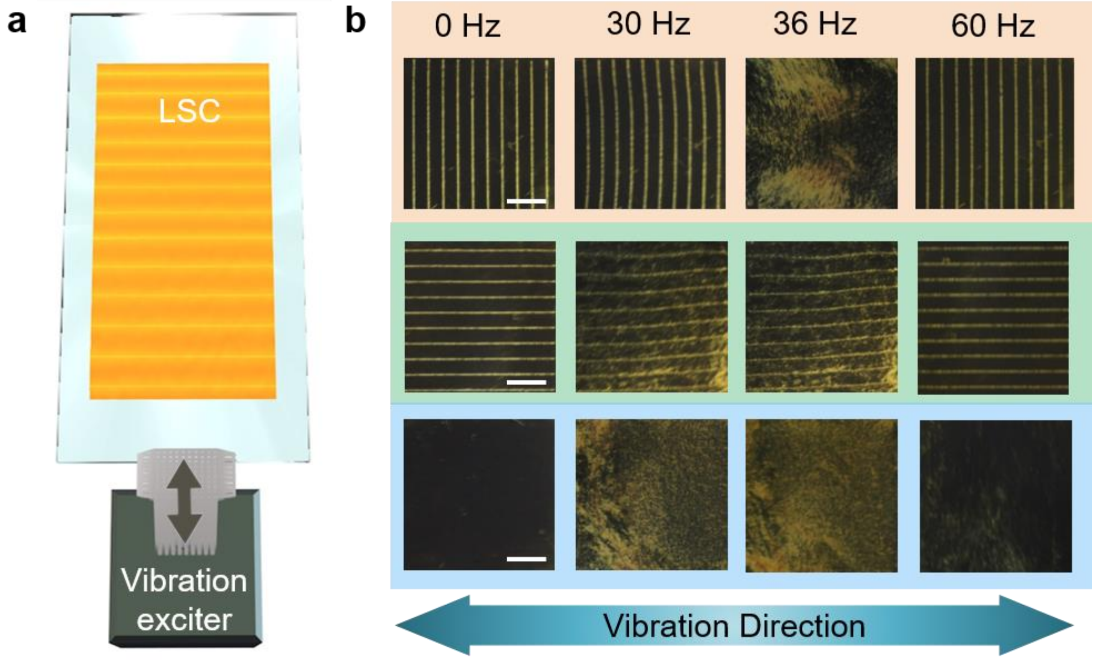

Supplementary Figure 16. Vibration tests of LMCs. a, Schematic of the forced vibration experiments for LMCs. LMC (0.3 wt.%) is constructed in a glass cuvette and fixed to an electromagnetic vibration exciter. The tracking by POM evaluates the lattice integrity under vibrations with different frequencies. **b,** POM images for LMCs with reoriented domains arrange perpendicular (top row), parallel (middle row) to the vibration direction and GO LC without supra lattice (bottom row) after a 2-min vibration at various frequencies, Scale bars, 500 μm.

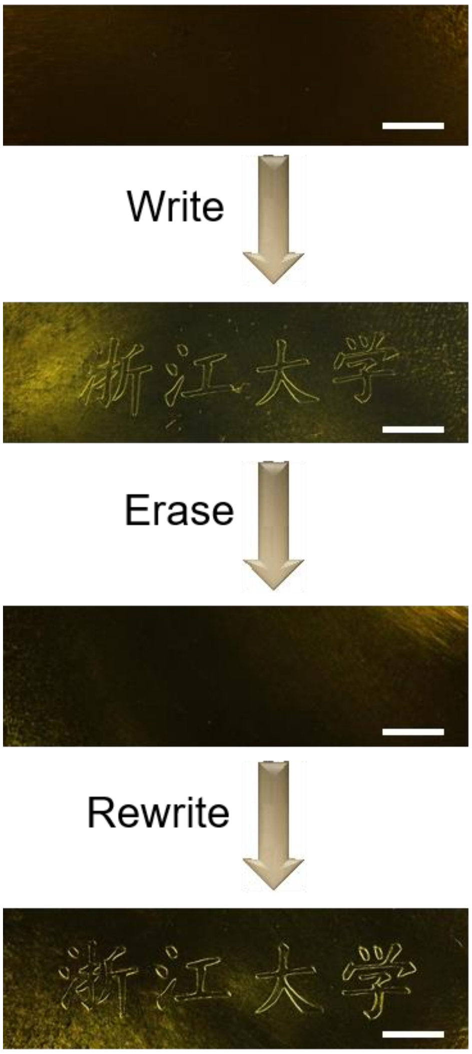

Supplementary Figure 17. The repeatability of SML. The write, erase and rewrite processes show the repeatability of the SML, Scale bar, 2 mm. Specifically, the write process is achieved by a typical SML process described above; And the erase process is realized by manual vibration for a few seconds to erase the existing patterns and followed by a blade casting process to offer LCs with horizontal arrangement. After that, the LCs is prepared for a rewriting process.

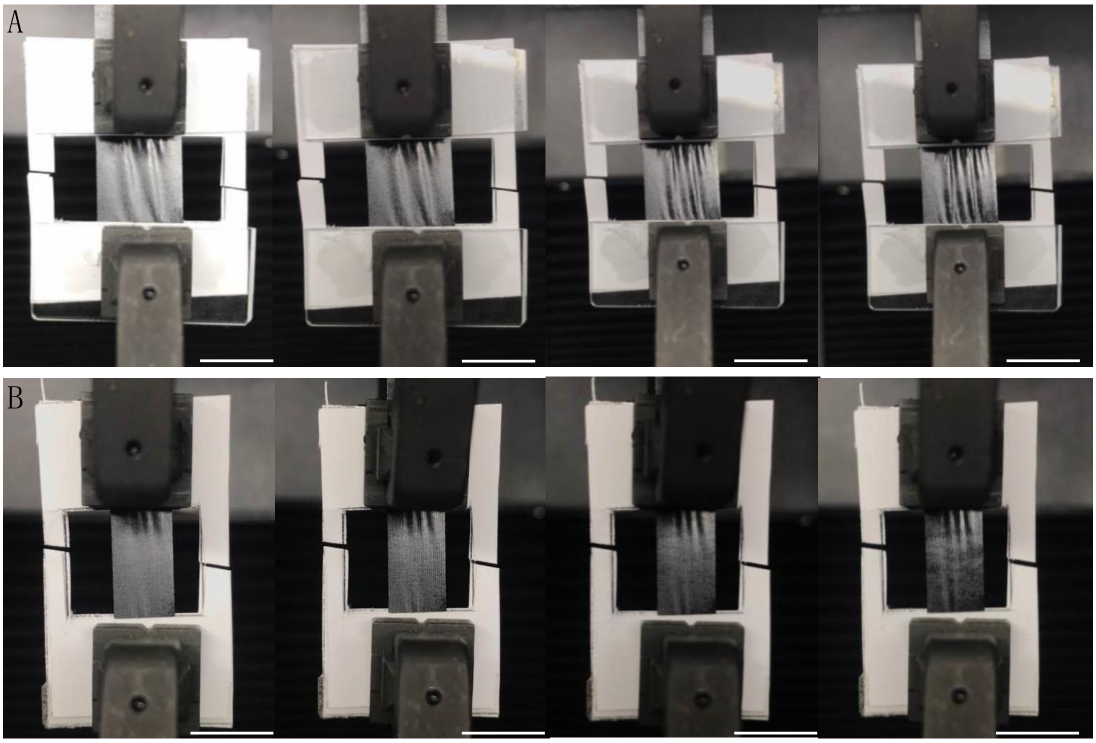

Supplementary Figure 18. The images of smooth and patterned RG films in mechanical tests. **a,** Images of smooth RG film in mechanical tests. Severe buckling generated with increasing strain due to the intense stress concentration, which was adverse to the mechanical performance of RG film. **b,** Images of patterned RG film in mechanical tests. Under small strain, no obvious buckling was observed. When the strain is great enough, slight buckling came into being, Scale bar, 1 cm.

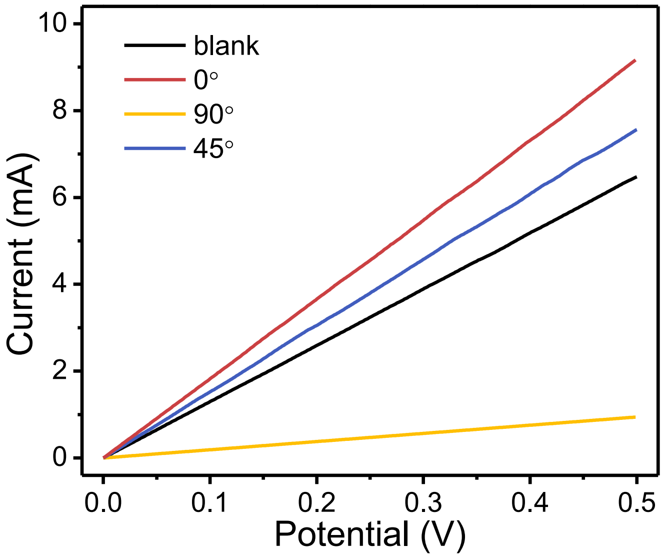

Supplementary Figure 19. The electrical conductivity of patterned RG films. The electrical conductivity varies dramatically with different angles between the direction of the ridge-like structure and the direction of the current. When the ridge-like structures arranged parallel to the direction of current, the film showed higher conductivity (~ 6385 S m^{-1}), even higher than the smooth RG film (~ 5742 S m^{-1}), which could contribute to the higher orientation of graphene sheets. On the other hand, when the ridge-like structures were vertical to the direction of current, these ridges would act as obstacles for the current. As a result, the conductivity of film decreased to about 761 S m^{-1}.

Supplementary Table

	0.01 wt.%	0.03 wt.%	0.06 wt.%	0.09 wt.%	0.3 wt.%
τ_{f}	48	203	527	4555	——
τ_{S}	10.4	52	137	2582	——

Supplementary Table 1. Fitted relaxation time for different LMCs. Half relaxation time calculated from the *f*-*t* curves (τ_f) and *S*-*t* curves (τ_S) for LMCs with different concentrations. The corresponding τ_S is smaller than τ_f, implying that the translational order of the supra crystal lattice relaxed faster than reoriented grain itself. For LMCs of 0.3 wt.%, no visible relaxation was observed in a 30-day-long experiment.

Supplementary Movie Legends

Supplementary Movie 1. Fabrication of LMC with p6mm symmetric lattice.

Supplementary Movie 2. Fabrication of LMC with 8mm symmetric lattice.

Supplementary Movie 3. Fabrication of LMC with director map of handwriting.
